# Assessment of patients’ dental anxiety levels in the context of infectious diseases: development and validation of Musa Kazim’s Dental Anxiety Scale (MK-DAS)

**DOI:** 10.1186/s40359-023-01516-5

**Published:** 2024-01-18

**Authors:** Musa Kazim Ucuncu, Merve Yildirim Ucuncu

**Affiliations:** 1 Altinbas University, Faculty of Dentistry, Department of Restorative Dentistry, Istanbul, Turkey; 2 Istanbul University, Institute of Graduate Studies in Health Sciences, Istanbul, Turkey

**Keywords:** COVID-19, Dental anxiety, Infectious disease, Surveys and questionnaires, Survey methodology

## Abstract

**Objectives:**

The study aimed to develop and validate a new scale called Musa Kazim’s Dental Anxiety Scale (MK-DAS) to measure dental anxiety in relation to infectious diseases.

**Methods:**

The study utilized a cross-sectional design and recruited participants from Faculty of Dentistry, Altinbas University. The sample included 289 participants who were seeking dental treatment. The Modified Dental Anxiety Scale (MDAS) was employed for the purpose of assessing levels of dental anxiety. In contrast, the MK-DAS, comprised a series of seven inquiries specifically targeting concerns regarding the treatment procedure and the fear of contagion. The data was analyzed using various statistical methods, including descriptive statistics, exploratory factor analysis, criterion validity, cluster analysis for cut-off points, and test-retest reliability.

**Results:**

The factor analysis revealed that MK-DAS had a two-factor structure. The first factor consisted of five items related to various aspects of the treatment process (α:0.837), while the second factor included two items related to the fear of infectious diseases (α:0.747). The scale showed good reliability, as indicated by high Cronbach’s alpha coefficients for both factors. Strong positive correlations were found between MDAS and the first factor of MK-DAS (r = 0.857; *p* < 0.01), moderate positive correlations between MDAS and the second factor (r = 0.323; *p* < 0.01), and a strong positive correlation between MDAS and the overall of MK-DAS (r = 0.782; *p* < 0.01). Additionally, the cluster analysis yielded a cut-off score of 17 based on the k-means analysis. Moreover, test-retest reliability analyses indicated that dimension 1 (ICC: 0.904), dimension 2 (ICC: 0.840), and overall MK-DAS (ICC: 0.944) demonstrated high internal consistency.

**Conclusion:**

The MK-DAS is an innovative and modern dental anxiety scale that has been proven to be reliable and valid, surpassing the comprehensiveness of the MDAS.

**Supplementary Information:**

The online version contains supplementary material available at 10.1186/s40359-023-01516-5.

## Introduction

Dental anxiety, more commonly referred to as odontophobia, represents a prevalent challenge within the field of dentistry. This condition not only places constraints on a dentist’s treatment options but also presents considerable difficulties for the patient [1]. The quality of life and daily existence for individuals grappling with severe dental anxiety is significantly compromised. The deterioration of their oral and dental hygiene becomes a major source of concern, eroding their self-esteem. Moreover, this anxiety can often be accompanied by severe psychiatric and psychosomatic conditions, including social isolation, despondency, as well as a reluctance to seek necessary medical care and adhere to proper oral hygiene practices [2, 3].

The swift propagation of SARS-CoV-2 (severe acute respiratory syndrome coronavirus-2) in the early months of 2020, better known as COVID-19 (coronavirus), became a reality. Due to its ability to spread through airborne droplets, COVID-19 can easily be transmitted between patients and physicians or vice versa, making dentists the professional group most susceptible to COVID-19 transmission [4]. Furthermore, when compared to COVID-19, Influenza, which has been recognized as a pandemic in different periods and has caused up to 600,000 deaths worldwide, can also lead to severe consequences [5]. The oral cavity, with its rich secretion and vascularization, provides a favorable environment for infectious diseases, posing a continuous risk to dentists and their patients, especially when they deviate from their standard sterilization and hygiene protocols [6]. Thus, studies have substantiated that the fear and anxiety levels of physicians have surged in the face of the potential contagion of virulent diseases like COVID-19 [7].

Numerous studies, spanning various departments of the medical field, have endeavoured to quantify the levels of anxiety experienced by both physicians and patients in the face of the ubiquitous threat posed by COVID-19 [8, 9]. Given that the very possibility of contracting this pathogen is a significant source of anxiety in and of itself, it is inconceivable that this reality wouldn’t engender a commensurate increase in dental anxiety levels. In the realm of dentistry, metrics such as Corah’s Dental Anxiety Scale (CDAS) [10], Modified Dental Anxiety Scale (MDAS) [11], and Dental Fear Survey (DFS) [12] have been developed to quantify levels of dental anxiety in patients. CDAS, developed by Corah and colleagues [10] for the purpose of evaluating dental anxiety, has proven to be a dependable, valid, and advantageous tool in predicting a patient’s level of distress in the dental setting. However, it has been subject to criticism for its inability to encompass all dimensions of dental phobia [13]. MDAS offers a more straightforward method of answering and includes an added inquiry concerning the administration of local anesthetic [11]. Not only does the MDAS incorporate questions about conventional treatment, it also possesses the added benefit of being a time-efficient and user-friendly assessment tool. The MDAS was developed by Dr. Humphris by adding a question related to injection fear to the CDAS [11], and over the years, its validity and reliability have been established through studies conducted on patients in various countries [14–16]. Furthermore, the included MDAS in this study is a scale that has been translated into Turkish, and its reliability and validity have been demonstrated [17, 18], making it a scale that has been utilized in studies conducted in our country [3, 19].

In light of this information, it is known that these mentioned questionnaires are inadequate in measuring the anxiety that individuals may develop or experience regarding the possibility of contracting infectious diseases such as COVID-19, HIV, Influenza, etc., in a dental clinic environment. These questionnaires do not include questions specifically related to this concern, and as a result, there is a possibility of incomplete and erroneous interpretation of dental anxiety in this context.

When developing a new scale, adhering to numerous criteria and standards is crucial to achieve high levels of validity and reliability. Utilizing a scale with inadequate validity leads to conducting analyses with low statistical power, while using a scale with low reliability introduces bias and results in erroneous data [20]. For a new scale, the initial steps involve conducting a literature review, determining the measurement format (e.g., Likert-type scale), ensuring clear and comprehensible items, seeking expert opinions, conducting pilot or pre-test studies, and finally evaluating validity and reliability through statistical analyses [20, 21]. Based on these principles, the aim was to build upon the Modified Dental Anxiety Scale (MDAS), which is one of the most commonly used and easily understandable dental anxiety scales in dentistry literature, and adapt it to the trend. To the best of our knowledge, no study has yet been conducted in the literature to assess dental anxiety levels specifically related to the possibility of contracting infectious diseases. In this context, the adaptation of MDAS by adding a question specifically related to this aspect was not suggested by Dr. Humphris; instead, he made a comment that developing a novel scale would be more appropriate and accurate similar to MDAS. As a result, we selected the MDAS as the most appropriate scale for measuring dental anxiety and contacted Dr. Humphris for his expert advice and recommendations. Following Dr. Humphris’s guidance, we developed a novel scale called the Musa Kazim’s Dental Anxiety Scale (MK-DAS) and used it to determine dental anxiety levels in our study, while also investigating criterion validity with the MDAS. In the present study, the validity and reliability of MK-DAS were assessed. The hypothesis of our study posits that the MK-DAS is a valid and reliable anxiety scale, concurrently demonstrating correlation with the MDAS.

## Materials and methods

### Sample design

This research, conducted through face-to-face questionnaire forms, was designed as a cross-sectional study. The study recruited participants among those seeking dental treatment at Faculty of Dentistry, Altinbas University. This study, encompassing a three-part questionnaire, commenced subsequent to receiving approval from the Ethics Committee (File No: 2022/68–22). Prior to filling out the aforementioned forms, participants were required to complete an Informed Voluntary Consent Form, which signified their agreement to partake in the study. The survey utilized in this study was composed of three distinct parts. The first part consisted of inquiries aimed at assessing sociodemographic variables such as age, gender, and educational background. Due to the hospital’s location in the Bakirkoy district which ranks in the first tier of the socioeconomic development index, it can be stated that this study comprises individuals residing in the Bakırköy district and its surrounding areas, with their socioeconomic level exceeding a certain threshold [22]. The second part encompassed the MDAS, which was adapted from CDAS by Dr. Humphris [11], previously translated into Turkish, and its reliability and validity have been established [17, 18]**.** Lastly, the third segment included the MK-DAS questionnaire, which was developed in Turkish in accordance with the recommendations and approval of Dr. Humphris. This study, conducted at a single center, enrolled 289 participants, excluding those under the age of 18 and those who reported having a systemic disease, undergoing psychological treatment, or taking medication regularly within the last 6 months. In the process of scale development, there are varying opinions regarding the recommended sample size, ranging from at least 5 times [23], 10 times [24], to 15 times [25] the number of items. Taking into consideration the literature in this regard, particular attention was given to ensuring a sample size of at least 120, and the study was completed with 289 participants.

### MDAS

To assess dental anxiety levels, the Modified Dental Anxiety Scale (MDAS) was employed, which was derived from Dr. Corah’s Dental Anxiety Scale by the addition of a question related to injections by Dr. Humphris [11]. The MDAS which was translated into Turkish and its validity and reliability were established [17, 18] encompasses the following inquiry items: “(S1) In the event that you visit the dentist tomorrow, (S2) When you sit in the waiting area, (S3) During the use of sharp dental instruments to treat your tooth, (S4) While having your teeth cleaned of plaque, and lastly (S5) in the event of an injection, how would you feel?“. The response options for each question include the categories of “Extremely afraid,” “Very afraid,” “Afraid,” “Less afraid,” and “Not afraid.” In the MDAS, a Likert-type scale is employed, where each option from “Not afraid” to “Extremely afraid” is assigned a score from 1 to 5, with “Not afraid” being assigned 1. The lowest possible score on the survey is established as 5, whereas the highest possible score is 25. The threshold score for determining high dental anxiety was established as (≥19), whereby respondents who scored above this value were deemed to exhibit elevated levels of dental anxiety.

### MK-DAS

The Musa Kazim’s Dental Anxiety Scale (MK-DAS) comprises eight questions designed to delve into the chronology of the treatment process and reflect modern fear trends (refer to the document titled MK-DAS English version). The questions are as follows: 1) “How do you feel when you are on your way to your appointment?” 2) “How do you feel while waiting in the clinic’s waiting room for your dentist to call you in?” 3) “How do you feel about the cleanliness and sterility of all the devices and materials that will be used during the procedure?” 4) “How do you feel when you see the dentist holding a needle for the first time, just before the treatment starts?” 5) “How do you feel when the dentist works with noisy instruments and rotating tools inside your mouth?” 6) “How do you feel when the dentist works with silent hand instruments inside your mouth? 7) “How do you feel about the possibility of contracting infectious diseases namely COVID-19, Hepatitis B, Influenza, etc. in the clinical environment or during the treatment?“ 8) “How do you feel about the possibility of experiencing complications, sensitivity, pain, swelling, bleeding, etc., which may require you to revisit the dentist shortly after the procedure?“. The survey was constructed using a likert-type scale, where each option was assigned, a score ranging from 1 to 5, with 1 representing “any fear, relaxed“ and 5 indicating “intense fear”. The minimum possible score was set at 8, while the maximum attainable score was capped at 40. To ensure the utmost precision and dependability in gauging levels of apprehension, various statistical analyses and methods were used to determine the validity and reliability of the scale. The optimal threshold value was computed and its association with the MDAS was assessed using rigorous statistical analysis. The commencement of the study was preceded by a preliminary trial that encompassed a group of 50 participants. The results of the pre-test served as a cornerstone in the formulation of the ultimate version of the measurement instrument. As a prelude to the pre-test, specialists were consulted during the developmental phase of the scale.

### The statistics analysis

The data were statistically analysed using IBM SPSS software (Chicago Inc., USA, version 21). Descriptive statistics such as mean, standard deviation, and frequencies were used. Exploratory factor analysis was conducted to determine the construct validity of the scale, and Cronbach’s alpha reliability coefficients were calculated to determine the scale’s reliability. The cut-off value was determined. To assess the distribution of MDAS and MK-DAS scores according to gender, Q-Q Plots and Skewness-Kurtosis normality tests were applied, and based on the results obtained, t-tests were utilized.

#### Exploratory factor analysis (EFA)

To determine whether the scale was suitable for factor analysis, both the Kaiser-Mayer-Olkin (KMO) test and the Bartlett test were conducted. The KMO coefficient was calculated to test the sample size, and normal distribution of the population was expected for factor analysis, which was examined using the Bartlett test. Specifically, a KMO test result close to 1.000 and a statistically significant result for the Bartlett sphericity test were expected. In determining the total number of factors in the scale, a Scree Plot graph was used, which displays the eigenvalues’ scatter plot, along with the proportion of the explained variance. The process of assigning or removing scale items from the factors in factor analysis involved examining the factor loading values. Factor loading value is a coefficient that describes the relationship between the items and the factors It was expected that the load values of items in their respective factors would be high. If the factor load of any item was less than 0.30 or if the difference between the factor loads of the item in two different factors (cross-loading) was less than 0.10, the item was removed from the scale and the analysis process continued. This decision was made to ensure the validity and reliability of the factor analysis.

#### Reliability analysis

The Cronbach’s alpha coefficient indicates the reliability level of the scale, ranging from 0 to 1. The reliability of the scale was interpreted based on the alpha (α) coefficient as follows [26]: 1) If .00 ≤ α < .40, the scale is not reliable. 2) If .40 ≤ α < .60, the scale has low reliability. 3) If .60 ≤ α < .80, the scale is fairly reliable. 4) If .80 ≤ α < 1.00, the scale is highly reliable.

#### Test-retest reliability

The consistency and stability of responses over time were examined using data collected from the same group (*n* = 20) at two different points in time (at 2 week intervals). Specifically, high ICC values and narrow confidence intervals were used to test the consistency of MK-DAS dimensions and overall scores between test-retest, and high internal consistency was expected. The sample size determination of 20 participants for the test-retest analysis was carried out following the recommendations set by Walter, with a significance level set at α = 0.05, β = 0.20, ρ0 = 0.1, ρ1 = 0.6, and *n* = 2 [27]. The inception point of MK-DAS, the initial phase of the study, the developmental and evaluational processes were depicted in the form of a flowchart (Fig. 2).

## Results

### Sociodemographic data

The mean age of the study group is 31.68 ± 14.40, with 37% male and 63% female participants. The mean age of women was 29.70 ± 12.69, whilst the mean age of men was 35.02 ± 16.42. Of the participants, 10.7% are high school graduates or below, 71.6% have a bachelor’s degree, and 17.6% have a graduate degree or higher (*n* = 289) (Table 1). According to the MDAS, women (10.22 ± 4.08) have a higher dental anxiety score compared to men (11.07 ± 4.45) (*p* > 0.05), while the opposite is true for the MK-DAS (respectively, 14.14 ± 4.96–14.00 ± 4.74) (*p* > 0.05) (Table 2).

**Table 1:** Sociodemographic data

		n	%
Sex	Male	107	37.0
	Female	182	63.0
Educational level	Secondary education	31	10.7
	Undergraduate	207	71.6
	Postgraduate	51	17.6

**Table 2:** The mean of dental anxiety according to gender

Gender		n	Mean	Std. Deviation	t	p
MK-DAS	Male	107	14.14	4.96	0.239	0.812
	Female	182	14.00	4.74		
MDAS	Male	107	10.22	4.08	−1.611	0.108
	Female	182	11.07	4.45		

*t* test

### Exploratory factor analysis

According to the factor analysis conducted for the scale, the KMO value was calculated as 0.832. Therefore, the sample size is suitable for factor analysis (KMO > 0.500). In the context of the Bartlett test, the X2 value was 895.785 and was found to be statistically significant (*p* < 0.05). Thus, the normal distribution condition has been met. Based on the results of the KMO and Bartlett tests, it can be concluded that the data is suitable for factor analysis.

**Fig. 1:**
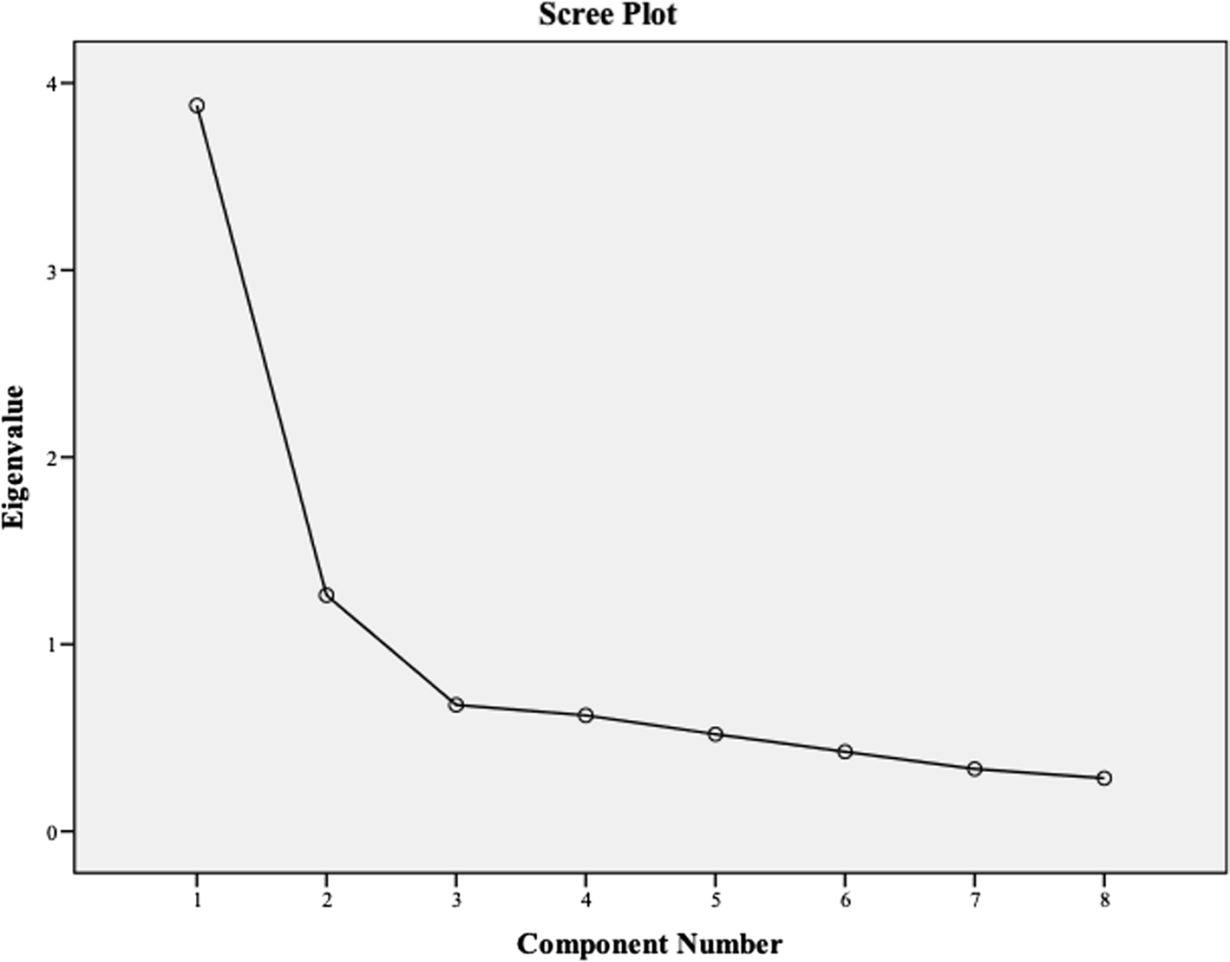
The determination of the two-factor structure & conducting varimax rotation analysis to determine the distribution of items across factors

In order to determine the factor structure of the scale, the Scree Plot graph showing the eigenvalue scatter was examined (Fig. 1). Upon examination of the graph, it was determined that the scale exhibited a 2-factor structure. To determine the distribution of questions into factors in the 2-factor structure, an analysis was performed with a factor count of 2 and a varimax rotation, and the distribution of questions and factor loadings are presented in Table 3.

**Table 3:** The distribution of questions and factor loadings

	Factor 1	Factor 2	Explained Variance Ratio	Cronbach’s Alpha
MK-DAS 2	0.872		43.576	0.837
MK-DAS 6	0.783			
MK-DAS 1	0.756			
MK-DAS 5	0.734			
MK-DAS 4	0.711			
MK-DAS 7		0.893	23.742	0.747
MK-DAS 3		0.860		

Within the scope of the study, the 8th item of the scale was removed from the analysis due to its low factor loading (redundancy). The distribution of the remaining 7 items was determined and presented in the table. The first factor of the scale consists of 5 items with factor loadings ranging from 0.711 to 0.872. The total variance explained by this factor is 43.576%, and its Cronbach’s Alpha coefficient is calculated as 0.837. The second factor of the scale consists of 2 items with factor loadings ranging from 0.860 to 0.893. The total variance explained by this factor is 23.742%, and its Cronbach’s Alpha coefficient is calculated as 0.747 (Table 3).

### The criterion validity MDAS and MK-DAS

The results of the criterion validity test conducted to examine the relationship between MDAS and MK-DAS are presented in Table 4. According to the analysis results, there is a strongly positive correlation (r = 0.857; *p* < 0.05) between MDAS and MK-DAS/Dimension 1, a moderately positive correlation (r = 0.323; *p* < 0.05) between MDAS and MK-DAS/Dimension 2, and a strongly positive correlation (r = 0.782; *p* < 0.05) between MDAS and MK-DAS (Table 4).

**Table 4:** Examining the criterion validity between MDAS and MK-DAS

		MK-DAS Dimension 1	MK-DAS Dimension 2	MK-DAS Overall	MDAS Overall
MK-DAS Dimension 1	r	1	.377^**^	.913^**^	.857^**^
	p		0.000	0.000	0.000
	n	289	289	289	289
MK-DAS Dimension 2	r		1	.723^**^	.323^**^
	p			0.000	0.000
	n		289	289	289
MK-DAS Overall	r			1	.782^**^
	p				0.000
	n			289	289
MDAS Overall	r				1
	p				
	n				289

**p < 0.01

### Cluster analysis for cut-off

In the study, cluster analysis using the k-means method was performed to determine the cut-off point for MK-DAS. As a result of the analysis, the cut-off value was determined as 17. Cluster 1 consisted of 78 participants with an MK-DAS score of 17 or higher, while Cluster 2 consisted of 211 participants with an MK-DAS score below 17. The mean MK-DAS score for Cluster 1 was 20.64, while the mean score for Cluster 2 was 11.62. There was a significant difference in MK-DAS scores between Cluster 1 and 2 (*p* < 0.05) (Table 5).

**Table 5:** Comparing cluster 1 and cluster 2 based on the cut-off value

		n	Mean	Sd	t	p
MK-DAS Overall	Cluster 1	78	20.64	3.22	22.497	0.000
	Cluster 2	211	11.62	2.43		

The mean MK-DAS score for Cluster 1 participants is 20.64, while the mean MK-DAS score for Cluster 2 participants is 11.62. There is a significant difference in MK-DAS scores between Cluster 1 and Cluster 2 (*p* < 0.05)

### Test-retest reliability

**Fig. 2:**
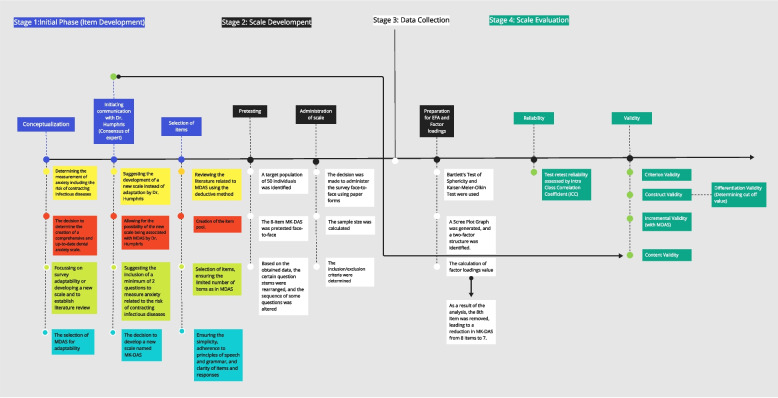
An illustrative flowchart depicting the emergence of MK-DAS. (Abbreviations: MDAS:Modified Dental Anxiety Scale, MK-DAS:Musa Kazim’s Dental Anxiety Scale, EFA:Exploratory Factor Analysis)

The consistency and invariance of responses over time were examined using data collected from the same group at two different time points. The ICC values obtained for MK-DAS Dimension 1 were excellent (ICC: 0.904, 95% confidence interval: 0.756–0.962), for MK-DAS Dimension 2 were quite good (ICC: 0.840, 95% confidence interval: 0.597–0.937), and for MK-DAS Overall were excellent (ICC: 0.944, 95% confidence interval: 0.859–0.978) (*p* < 0.001) (Table 6). Based on these findings, it was concluded that the MK-DAS is consistent over time Fig. 2.

**Table 6:** Test-retest reliability

	ICC	Lower Bound	Upper Bound	Value	p
MK-DAS Dimension 1	**0.904**	**0.756**	**0.962**	**10.372**	**0.000**
MK-DAS Dimension 2	**0.840**	**0.597**	**0.937**	**6.263**	**0.000**
MK-DAS Overall	**0.944**	**0.859**	**0.978**	**17.978**	**0.000**

## Discussion

The hypothesis that MK-DAS is a reliable and valid anxiety scale, and that it exhibits a positive correlation with MDAS, has been accepted based on the data obtained. Based on the MDAS results, females had higher dental anxiety scores compared to males; however, in the case of MK-DAS, the opposite was observed. Nevertheless, both scales did not show statistically significant differences in dental anxiety between females and males (*p* > 0.05). Studies in the literature have reported conflicting results, with some indicating higher scores in females compared to males [3, 19], while others report the opposite [28]. In our study, the lack of statistically significant differences in dental anxiety scores between genders in both scales and the close resemblance of dental anxiety scores between female and male individuals demonstrate, in accordance with the principle of criterion validity targeted in the hypothesis, that both scales are capable of making similar measurements.

Two of the criteria considered in patient selection were the absence of psychological and systemic disorders in individuals. Oral health implies the absence of disease and disorders in oral, dental, and craniofacial tissues, indicating that oral health is essentially interconnected with overall health and forms an inseparable entity [29]. Systemic diseases may present oral manifestations, and many oral diseases can also contribute to the deterioration of systemic conditions. Diseases observed in oral and dental health due to systemic conditions threaten individuals’ general health in the long term, leading to disruptions in their nutrition, limitations in social life, and decreased self-confidence. All of these factors can lead to a decline in life standards psychologically and can push individuals into psychological distress [30]. Furthermore, to ensure standardization among the participants and minimize potential biases in responses from individuals with psychological disorders or medication use, only individuals without systemic and psychological disorders and with good general health were included in the study [31]. To reduce the complexity caused by medication use and to render the data more transparent, individuals who had not used any medication in the last 6 months were included to neutralize the possible effects of medication.

When a new concept emerges and there is no existing model to measure the underlying structure or innovation, researchers may embark on the process of developing a new scale. The aim is to create a novel scale that measures the focal variable and captures the innovation. While there are various scales in the literature related to the subject, a decision can be made to develop a new scale from a different perspective [32]. However, instead of developing a completely new scale, modifying or adapting an existing scale may appear to be a quicker and more straightforward approach [33]. In the literature, several scales are available for measuring dental anxiety, and among them, it was determined that the Modified Dental Anxiety Scale (MDAS) was the most suitable choice for our study’s objectives, sample group, and psychometric properties. Based on this decision, it was decided to adapt the MDAS, and communication was established with the scale’s owner, Dr. Humphris. Initially, it was planned to modify the scale by adding a question similar to the 7th question of the MK-DAS to the MDAS. However, upon the recommendation of Dr. Humphris, this plan was abandoned. The focus was on developing a new scale, and following Dr. Humphris’s recommendation, it was aimed to have at least 2 questions in the new scale to measure anxiety related to contracting infections. Then, the redesigned MK-DAS was aimed to be composed of as few questions as possible. Survey fatigue poses a serious challenge for researchers in survey studies [34]. Shorter surveys have been consistently regarded as superior to longer surveys [35]. In paper-based face-to-face surveys, and particularly in lengthy and multi-sectioned surveys such as DFS and IDAF-4C+, survey fatigue can be observed in actions such as consistently marking the same answer in bubbles throughout all pages or giving similar responses to consecutive questions. The existence of “double-barreled” questions, which include two separate questions within a single question, in the IDAF-4C+ questionnaire affects the responses given. Moreover, online surveys are susceptible to issues such as the possibility of the browser being closed before the completion of the survey and the tendency to provide the same answer for several questions due to survey fatigue [35, 36]. Therefore, in this study, a scale that resembles MDAS in terms of format, is slightly more comprehensive than MDAS but has a limited number of questions, was developed by considering the ease and speed of completing MDAS; and whether the two scales correlate with participants was investigated.

When designing a survey, the objectives of the subject being researched determine the starting point of the process. After determining the content of the survey, the second stage involves deciding on the method of implementation. The third stage involves the development of the survey form, in which the questions are put into words, the order of the questions is determined, and the answer options are established as the basis [37]. During this stage, the questions are articulated in words, and the order of the questions and the response options are determined. The first stage of survey design involves adapting the surveys to modern trends and the current era. In this stage, a roadmap was established by seeking expert opinions from Dr. Humphris. The second stage involved the decision to administer the survey face-to-face, and it was determined that responses would be collected from patients visiting our university hospital for dental treatment. In the third stage, a pre-test was conducted, and the content of the questions, the question format, and the order of the questions were arranged based on the data obtained. It has been noted that some of the problems encountered in survey research are related to pre-testing. These include conducting pre-tests with a small number of participants [38], rushing through the process in an unstructured manner to save time [39], and often applying the pre-test to students who are not part of the target audience [40]. Pre-tests allow for the evaluation of variability in responses, difficulties in answering, and the participant’s interest in the questions. Pre-tests are useful in determining the flow of the survey questions. Based on this information, our study conducted a pre-test with 50 participants [41] using the respondent debriefing method [42]. Through this practice, respondents are allowed to interpret survey questions and after completing the survey, follow-up questions are asked to determine whether the survey and questions were correctly perceived. Thus, interpretation errors and difficulties in answering were identified. Based on the data obtained, the order of some questions was rearranged, certain question stems were expressed more clearly, and phrases that made the survey easier for participants to understand were included.

According to the 8-item MK-DAS (As a consequence of the factor analyses, the 8th item was omitted from the study and survey scope due to redundancy, leading to the continuation of the scale with 7 items), dental fear is considered to be a variable phenomenon that can vary depending on the conditions and environment. In creating the scale, not only the MDAS framework was utilized, but also the above-mentioned factors were considered. Since dental fear can vary based on emotional states, the most accurate measurement of dental fear should encompass the entire treatment process and should not include time periods prior to treatment. The crucial moment when treatment begins is when individuals first confront their fear, that is, when they embark on the journey of treatment. Therefore, instead of asking the question “How would you feel if you were to go to the dentist tomorrow?” the scale begins with the question “How do you feel when you embark on the journey of treatment?” Responses given in a more comfortable environment away from dental clinic settings and in different states of mind may differ from those given while sitting in the dentist’s chair [3, 43]. According to a study by Alghareeb et al., 42.9% of participants showed very low levels of anxiety in response to the first question of the MDAS [44]. For example, individuals who complete the MDAS survey just before undergoing treatment may not respond to the first question in the same way as they would in the comfort of their own home. This situation can also be considered applicable to the other questions on the test. Additionally, a study conducted by Üçüncü et al. found that the frequency of dental anxiety was much lower in online surveys compared to in-person surveys [3]. In summary, when measuring dental fear levels, directing questions towards the treatment process more accurately may provide a more accurate assessment of fear levels. The questions posed to participants in the MK-DAS focus on important aspects of the treatment process. The questionnaire presents questions to the individual in a specific chronological order. The start of treatment is actually the beginning of the treatment process, and the anxiety associated with waiting in the clinical setting until the physician takes the patient, waiting in the same environment as other patients and sharing similar surfaces, tools, etc. is separately and sequentially measured. Waiting for long periods in the clinic can increase patients’ anxiety. One of the first and most significant fears that the patient will encounter when taking their place in the dental chair is the fear of injection. Depending on the nature of the treatment to be performed, the injection question has been brought forward in the MDAS questionnaire due to the injection that will be administered before starting the treatment, followed by the use of noisy dental instruments. Not only rotating instruments but also non-rotating and noiseless hand instruments can be among the causes of dental fear. According to literature [44], extraction is one of the most feared procedures and results in higher levels of dental anxiety compared to other procedures such as restoration, root canal treatment, and periodontal scaling. Therefore, a question has been prepared to measure the level of dental fear that may arise due to noiseless hand instruments.

Fear has been defined as a state of agitation and alarm arising from the presence or perception of a sudden danger [45]. While Farmilant suggests there are about 25 types of fear [46], the type of fear expressed as dental or dentist phobia can be associated with Farmilant’s “unknown fear”. Fear of the unknown is not based on logic. It is related to the feeling of danger that certain events may occur [46]. The literature indicates that the fear that arises especially in early ages has a complex psychological structure, and that different methods from modern and conventional approaches need to be applied to understand it. Accordingly, a study was conducted on this issue [47]. The level of fear in young individuals, which is traditionally measured using conventional methods, does not always enable them to express things they find difficult to put into words. In this study that utilized drama as a method, a young child’s upcoming dental appointment triggered an “unknown fear” within them because the main character does not know what to expect [47]. In this context, an adult who previously had little or mild dental anxiety when visiting the dentist may experience a fear of the unknown when going to the dentist during the COVID-19 pandemic, just like a small child who doesn’t know what to expect. They may feel a significant amount of anxiety about contracting COVID-19 and sit in the chair with a different level of fear. One of the objectives of developing MK-DAS is the absence of another dental anxiety scale that can measure this concern or the lack of modernized questions within existing scales to address this issue. There is currently no question in the dental fear measurement scales previously developed and present in the literature that detects concerns about the possibility of contracting infectious diseases such as COVID-19. Dentists and the patients they treat are at risk of infections transmitted through blood and saliva during daily treatment procedures [48]. Research has shown that many diseases in dentistry can be transmitted through direct and indirect contact, such as injuries caused by cutting and piercing tools, contact with contaminated instruments, devices, and surfaces, and settlement on damaged skin or mucous membranes [49, 50]. Among these diseases, herpes simplex types 1 and 2, staphylococcus, streptococcus, tuberculosis, Hepatitis B, Hepatitis C, and HIV infections are of significant concern in dentistry [51]. Therefore, it is inevitable that patients are worried about contracting these diseases. To address this concern, two questions assessing the fear of contracting infectious diseases was included in the design of MK-DAS (3rd and 7th questions). In order to accurately measure dental anxiety, the MK-DAS questionnaire attempted to assess anxiety throughout the entire treatment process and also the anxiety that might arise in the moments just before the treatment, which could be related to post-operative discomforts or complications that patients might experience after the treatment. With this purpose in mind, the 8th question was formulated. Individuals may postpone dental treatments and avoid the dentist due to the possibility of experiencing various post-operative discomforts (such as swelling, bleeding, dentin sensitivity, etc.) and complications immediately after or on the following day of the procedure. Based on clinical experiences, the 8th question was added to the MK-DAS and tested; however, due to the low values obtained in the factor analyses, this question was unfortunately subsequently removed from the analysis and the original survey.

The sphericity test, which is commonly encountered in factor analysis, is a test that is often overlooked in practice but is essential to be applied. Just as the homogeneity of variances is an important step in a variance analysis process, the sphericity test in factor analysis also serves the same mission. First, the data’s suitability for factor analysis is tested by checking sphericity, and if the sphericity test is statistically significant, factor analysis can proceed. This situation is referred to as the consistency of variables [52, 53]. Therefore, the MK-DAS scale was subjected to exploratory factor analysis. There are differences of opinion regarding the determination of the sample size [54]. Although it is commonly stated that the larger the sample size, the more reliable the results of factor analysis will be [55], there are disagreements about determining the appropriate sample size. It has been suggested that 10 or 15 participants per item can be included in the analysis [26, 55], and if the factor loadings are greater than 0.6, a minimum of 150 participants can be used [56]. Therefore, this study was conducted with a sample of 289 participants in accordance with the literature. In order for the sample to be considered sufficient, the Kaiser-Meyer-Olkin (KMO) coefficient should be at least 0.50; between 0.80 and 0.90, it is considered highly satisfactory, and > 0.90 is interpreted as excellent [57]. In our study, this value was calculated as 0.832, indicating that the sample size is rated as highly satisfactory.

Indeed, although validating a newly developed scale through validity and reliability analyses provides scientific evidence of its validity and reliability, it is common to also conduct correlation analyses with an already established and widely used scale. During the development of the scale, Exploratory Factor Analysis is employed to determine the dimensions of the scale. These dimensions, which are fundamental structural components, are used to examine the similarities and differences between the questionnaires [58, 59]. In this study, based on the results obtained after Exploratory Factor Analysis, it was presumed that the items constituting the MK-DAS represent a common feature or topic when combined. Accordingly, Dimension 1 (comprising questions 1, 2, 4, 5, and 6) and Dimension 2 (comprising questions 3 and 7) were formed. Subsequently, the correlation between these dimensions and the entire MK-DAS (referred to as “MK-DAS Overall”) and MDAS was analysed. The term “Overall” is typically used to assess the overall effectiveness or reliability of a scale. If there are high correlations between the dimensions and a high correlation with “Overall,” it indicates that the scale is reliable and valid across different dimensions. However, if there are low correlations between the dimensions and a high correlation with “Overall,” it suggests that there may not be enough variability among all the questions, and one dominant dimension may prevail. In this study, a strong positive relationship was observed between MK-DAS and MDAS regarding Dimension 1 and “Overall” (respectively r = 0.857; r = 0.782), and a moderately positive relationship was found for Dimension 2 (r = 0.323). This provides evidence of the accuracy of MK-DAS’s measurement target and its overall performance.

When developing a new scale, before collecting data from the main sample, a pilot study, known as test-retest, should be conducted with a small group having similar characteristics to the primary sample. This pilot study allows testing whether the questionnaire yields similar responses at different times in subsequent surveys, and it verifies the reliability of the scale. Test-retest involves collecting data from 20 to 30 individuals, as mentioned, at two different time points [60, 61]. Due to the fact that the time interval between the two administrations of the questionnaire can be determined based on the specific attribute being measured [62], in our study, a test-retest analysis was conducted with 20 participants, 2 weeks apart, for the MK-DAS. According to the study by Walter et al., numerous combinations were generated to construct exact power curves, and an extensive evaluation of the approximation was conducted. With the median value of the obtained *k*_approx_ - *k*_exact_ being 0.16 and the majority of these values being positive, it was argued that the approximation can be confidently utilized in the design of actual studies, and that the bias of this analysis is kept to a minimum [27]. Based on this, the values from the study and the generated table were utilized, leading to the decision of employing a sample size of 20 individuals for the test-retest analysis. The analysis results demonstrated that the MK-DAS dimensions and overall score exhibited high ICC values and narrow confidence intervals, thereby confirming strong consistency between repeated measurements and providing evidence of reliable measurement.

The cut-off value of a newly developed Likert-type scale is the threshold used for categorization, aiding in the determination of different groups or categories based on the scale scores [63]. This value is derived from the distribution of scores obtained from the scale. For instance, the lowest 25% and the highest 25% segments can be grouped separately for evaluation [63, 64]. Additionally, cluster analysis is employed to segregate individuals with similar characteristic profiles using the scale scores. Data from individuals with similarities are combined to form homogeneous groups. Determining the cut-off value becomes more facilitated after the formation of these homogeneous groups. Non-hierarchical, hierarchical agglomerative, or k-means algorithms are commonly used in cluster analysis [65–67]. These algorithms are instrumental in creating homogeneous groups by separating data based on similarities or distances. In our study, the k-means method was utilized to determine the cut-off value for MK-DAS, thereby obtaining Cluster 1 and Cluster 2. The analysis resulted in a cut-off value of 17, with 78 individuals having scores of 17 or above in Cluster 1, while 211 individuals had scores below 17 in Cluster 2.

One of the most significant limitations of this study is its restricted scope to Istanbul. It is necessary to conduct epidemiological studies involving multiple centers, categorizing different age groups and socioeconomic backgrounds, to assess dental anxiety using MK-DAS on a larger sample size. Additionally, further investigation can be conducted to examine the correlation of MK-DAS with various scales other than MDAS. Furthermore, in present study, individuals with a certain socioeconomic level above a threshold were included due to the location of the hospital. Consequently, the limitation arising from this context can be overcome in future research conducted among individuals from different regions or provinces with diverse socioeconomic backgrounds. Furthermore, it is recommended that the continuation of this study involves conducting structural model analysis using various techniques such as structural equation modeling (SEM) or confirmatory factor analysis (CFA) for MK-DAS.

## Conclusion

Anxiety levels related to dental procedures can be more accurately measured using surveys that are in line with current trends and the modern age. In present study, the MK-DAS questionnaire has been created for use by dental practitioners and clinicians. The factor analyses conducted have demonstrated significant interrelatedness among the questionnaire items, indicating their cohesiveness and internal consistency. Furthermore, the test-retest analysis conducted at different time points has confirmed the reliability and stability of the measurements. Notably, the MK-DAS questionnaire has exhibited a criterion validity with the established MDAS, providing evidence of its concurrent validity. These findings have important implications for clinical practice and dental education, as the MK-DAS questionnaire proves to be a valuable enstrument for assessing dental anxiety in a reliable and valid manner. The successful adaptation and validation of the MK-DAS questionnaire will foster a more nuanced comprehension and thorough assessment of dental anxiety. Consequently, it will elevate the caliber of clinician-patient rapport and yield notable advancements in the optimization of dental interventions.

### Supplementary Information

**Additional file 1.**

## Data Availability

There is no availability statement of data and materials in this manuscript.
