# Supplementary material for: Assessment of patients’ dental anxiety levels in the context of infectious diseases: development and validation of Musa Kazim’s Dental Anxiety Scale (MK-DAS)
Source: BMC Psychol. 2024 Jan 18;12:29. doi: 10.1186/s40359-023-01516-5 (PMC10795330; doi:10.1186/s40359-023-01516-5)
Supplement: Supplementary file 1 — Additional file 1. [file 40359_2023_1516_MOESM1_ESM.docx]

**MK-DAS (English Version)**

**1.How do you feel when you are on your way to your appointment?**

Any fear, relaxed.

Somewhat uneasy.

Tense.

Anxious and distressed.

Intense fear.

**2.How do you feel while waiting in the clinic's waiting room for your dentist to call you in?**

Any fear, relaxed.

Somewhat uneasy.

Tense.

Anxious and distressed.

Intense fear.

**3.How do you feel about the cleanliness and sterility of all the devices and materials that will be used during the procedure?**

Any fear, relaxed.

Somewhat uneasy.

Tense.

Anxious and distressed.

Intense fear.

**4.How do you feel when you see the dentist holding a "needle" for the first time, just before the treatment starts?**

Any fear, relaxed.

Somewhat uneasy.

Tense.

Anxious and distressed.

Intense fear.

**5.How do you feel when the dentist works with noisy instruments and rotating tools inside your mouth?**

Any fear, relaxed.

Somewhat uneasy.

Tense.

Anxious and distressed.

Intense fear.

**6.How do you feel when the dentist works with silent hand instruments inside your mouth?**

Any fear, relaxed.

Somewhat uneasy.

Tense.

Anxious and distressed.

Intense fear.

**7.How do you feel about the possibility of contracting infectious diseases namely COVID-19, Hepatitis B, Influenza, etc. in the clinical environment or during the treatment?**

Any fear, relaxed.

Somewhat uneasy.

Tense.

Anxious and distressed.

Intense fear.
